# Single-session upper limb plyometric training is as effective as two sessions for improving muscle strength, power, and serve velocity in male youth tennis players: a randomized parallel controlled study

**DOI:** 10.3389/fpsyg.2025.1539739

**Published:** 2025-01-27

**Authors:** Jian Wang, Qi Xu

**Affiliations:** ^1^Zhejiang College of Security Technology, Wenzhou, China; ^2^Gdansk University of Physical Education and Sport, Gdańsk, Poland

**Keywords:** resistance training, reactive strength, athletic performance, strength, power

## Abstract

**Introduction:**

Providing a balanced training stimulus that promotes positive adaptations without excessively increasing training volume—and thereby avoiding disruptions to the training process—is a challenging task for strength and conditioning coaches. This study aimed to compare the effects of one vs. two weekly sessions of upper limb plyometric training (ULPT) on muscle strength, power, and serve velocity in male youth tennis players.

**Methods:**

We conducted a randomized controlled study with two ULPT groups: one receiving one session weekly (ULPT1w) and the other two sessions (ULPT2w), alongside a control group maintaining regular tennis training. The intervention lasted 8 weeks. A total of 47 male youth tennis players (15.6 ± 0.9 years), competing at the trained/developmental level, participated in the study. Evaluations were conducted twice—once before the intervention and once after—assessing isometric shoulder rotation strength (internal [ISRT] and external [ESRT]) with a dynamometer, the medicine ball chest throw (MBCT), seated shot-put test (SST), and serve velocity using a radar gun.

**Results:**

In the post-intervention, significant differences were observed between groups for the variables of ISRT (*p* = 0.010; $\eta_{p}^{2}$ = 0.189), ESRT (*p* = 0.004; $\eta_{p}^{2}$ = 0.226), MBCT (*p* = 0.012; $\eta_{p}^{2}$ = 0.181), SST (*p* = 0.019; $\eta_{p}^{2}$ = 0.164), and tennis serve velocity (*p* = 0.004; $\eta_{p}^{2}$ = 0.226).

**Conclusion:**

The study found that ULPT significantly improves upper limb muscle strength, power, and serve velocity in young male tennis players, with both once and twice weekly training yielding similar benefits. As practical implications coaches can effectively incorporate ULPT once a week to enhance physical performance in young male tennis players.

## 1 Introduction

The tennis serve is crucial for overall match performance, as it not only initiates play with the potential to gain an immediate advantage through speed and placement but also sets the tone for the subsequent rally, influencing both offensive and defensive strategies (Gillet et al., 2009). Muscle strength and power are important factors in optimizing tennis serve and overall performance due to their direct influence on kinetic chain efficiency and stroke mechanics (Colomar et al., 2022b). Research indicates that greater muscle strength enhances the ability to generate force quickly, which is essential for the explosive movements required in a powerful serve (Colomar et al., 2023). Specifically, studies have shown that upper body strength, particularly in the shoulder and core muscles, correlates with serve velocity and accuracy (Palmer et al., 2018). Among these, the rate of force development (RFD) in shoulder internal rotation emerges as one of the most critical parameters (Baiget et al., 2022; Colomar et al., 2022a). Additionally, power output, defined as the rate at which work is performed, plays a crucial role in facilitating rapid racket acceleration and increasing ball speed, which in turn enhances overall performance (Kovacs and Ellenbecker, 2011). Consequently, developing targeted strength and power training regimens can significantly improve a player’s serve and competitive performance in tennis (Reid and Schneiker, 2008).

Among the various strength and power training methods available, plyometric training is particularly interesting because it can be performed in diverse settings without the need for substantial equipment (Deng et al., 2022). Research has established that plyometric training significantly contributes to positive adaptations in muscle strength and power across different sports, making it a valuable tool for athletes looking to enhance their performance (Booth and Orr, 2016). In particular, upper limb plyometric training (ULPT) can be particularly beneficial in supporting the development of tennis serve performance by potentially enhancing muscle strength and power through improved neuromuscular capabilities for generating explosive movements (Fernandez-Fernandez et al., 2016; Garcia-Carrillo et al., 2023).

Plyometric exercises, such as medicine ball throws and explosive push-ups, emphasize rapid stretching and shortening of muscles (Davies et al., 2015), which increases muscle potentiation and recruits fast-twitch fibers (Macaluso et al., 2012). Studies have shown that such training leads to greater improvements in upper body strength and power output—important components for an effective serve (Garcia-Carrillo et al., 2023). Additionally, plyometric training enhances the kinetic chain involved in the serve by promoting coordination and timing (Davies et al., 2015), which may translates into increased racket speed and ball velocity. Consequently, integrating upper limb plyometric exercises into a training regimen can establish a solid foundation for improvements in serve mechanics and overall tennis performance by maximizing the athlete’s explosive capabilities and functional strength (Deng et al., 2022).

However, the integration of ULPT must be carefully considered in relation to the player’s overall availability and the tennis training schedule, which often constrains adjustments and planning for coaches (Afonso et al., 2022). These challenges can be particularly pronounced for youth athletes, who must balance their training with numerous other responsibilities (e.g., academics, family). Therefore, minimizing the impact of strength training while ensuring its effectiveness becomes a key consideration. Previous studies on lower limb plyometric training have consistently showed that a single session can effectively enhance muscle strength and power in youth athletes across various sports, such as soccer (de Villarreal et al., 2008; Ramirez-Campillo et al., 2018; Bouguezzi et al., 2020). However, research on ULPT is particularly scarce (Deng et al., 2022). A recent systematic review (Deng et al., 2022) of plyometric training in tennis revealed a lack of studies on this topic and highlighted that none of the available research examined the impact of training volume, specifically the number of weekly sessions, on tennis players’ adaptations. Given this gap, it is essential to research the effects of different weekly training volumes of ULPT on key variables such as muscle strength, power, and serve velocity in tennis players.

Therefore, this study aims to compare the effects of one (ULPT1w) versus two (ULPT2w) weekly sessions of upper limb plyometric training (ULPT) on muscle strength, power, and serve velocity in male youth tennis players. We hypothesize that both ULPT1w and ULPT2w will significantly enhance muscle strength, power, and serve velocity compared to a control group. Furthermore, based on previous research on lower limb plyometric training (de Villarreal et al., 2008; Ramirez-Campillo et al., 2018; Bouguezzi et al., 2020), we anticipate that ULPT1w may be similarly effective as ULPT2w.

## 2 Methods

This study report follows to the CONSORT guidelines for reporting randomized study designs (Merkow et al., 2021).

### 2.1 Participants

The study included 47 male youth tennis players at the trained/developmental competitive level (McKay et al., 2022), with an average age of 15.6 years (±0.9) and a training history averaging 3.6 years (±0.8). Participants had a mean height of 170.0 cm (±6.1) and an average body mass of 58.7 kg (±7.2), resulting in a body mass index (BMI) of 20.3 kg/m^2^ (±1.9). Detailed characteristics of each group are listed in Table 1. Baseline comparisons of the demographic and anthropometric data for each group were conducted. No significant differences were observed between the groups in terms of age (*p* = 0.628), experience (*p* = 0.819), height (*p* = 0.104), or body mass index (*p* = 0.101). However, significant differences were found in body mass (*p* = 0.030), with players in the control group being significantly heavier than those in the ULPT1w group (mean difference: 6.3 kg; *p* = 0.030).

**Table 1:** Demographic and anthropometric data for the tennis players involved in this study.

	ULPT1w (*n* = 16)	ULPT2w (*n* = 16)	Control (*n* = 15)	Between-group ANOVA
Age (years)	15.4 ± 0.6	15.6 ± 1.0	15.7 ± 0.9	*p* = 0.628
Experience (years)	3.7 ± 0.9	3.7 ± 0.8	3.5 ± 0.6	*p* = 0.819
Height (cm)	169.3 ± 6.9	168.1 ± 6.0	172.6 ± 4.5	*p* = 0.104
Body mass (kg)	56.4 ± 6.2^¶^	57.4 ± 8.1	62.7 ± 5.8	*p* = 0.030
Body mass index (kg/m^2^)	19.6 ± 1.2	20.2 ± 1.9	21.1 ± 2.3	*p* = 0.101

ULPTw1: group participating in one session per week of upper limb plyometric training; ULPTw2: group participating in two sessions per week of upper limb plyometric training.

¶ Significantly different (*p* < 0.05) from control group.

These players regularly participated in regional tournaments, trained approximately three times a week, totaling around 330 min of tennis practice weekly. It is important to note that all training sessions were conducted exclusively by their respective coaches, without any involvement from the research team.

G*Power software (version 3.1.9, Universität Düsseldorf, Germany) recommended a total sample size of 9 participants to achieve a statistical power of 0.95 and a significance level of 0.05 for the *F* tests, specifically focusing on the repeated measures ANOVA for within-between interactions. This calculation was based on an effect size of 0.79, which was determined from the effect size observed in young tennis players who underwent ULPT related to serving velocity (Fernandez-Fernandez et al., 2016). With the required sample size determined, recruitment began by directly approaching the local tennis clubs, involving coaches and directors in the process. The research team outlined the study’s framework and extended a voluntary invitation to coaches, parents, and tennis players. Tennis players who expressed interest were then screened based on specific inclusion criteria.

**Figure 1:**
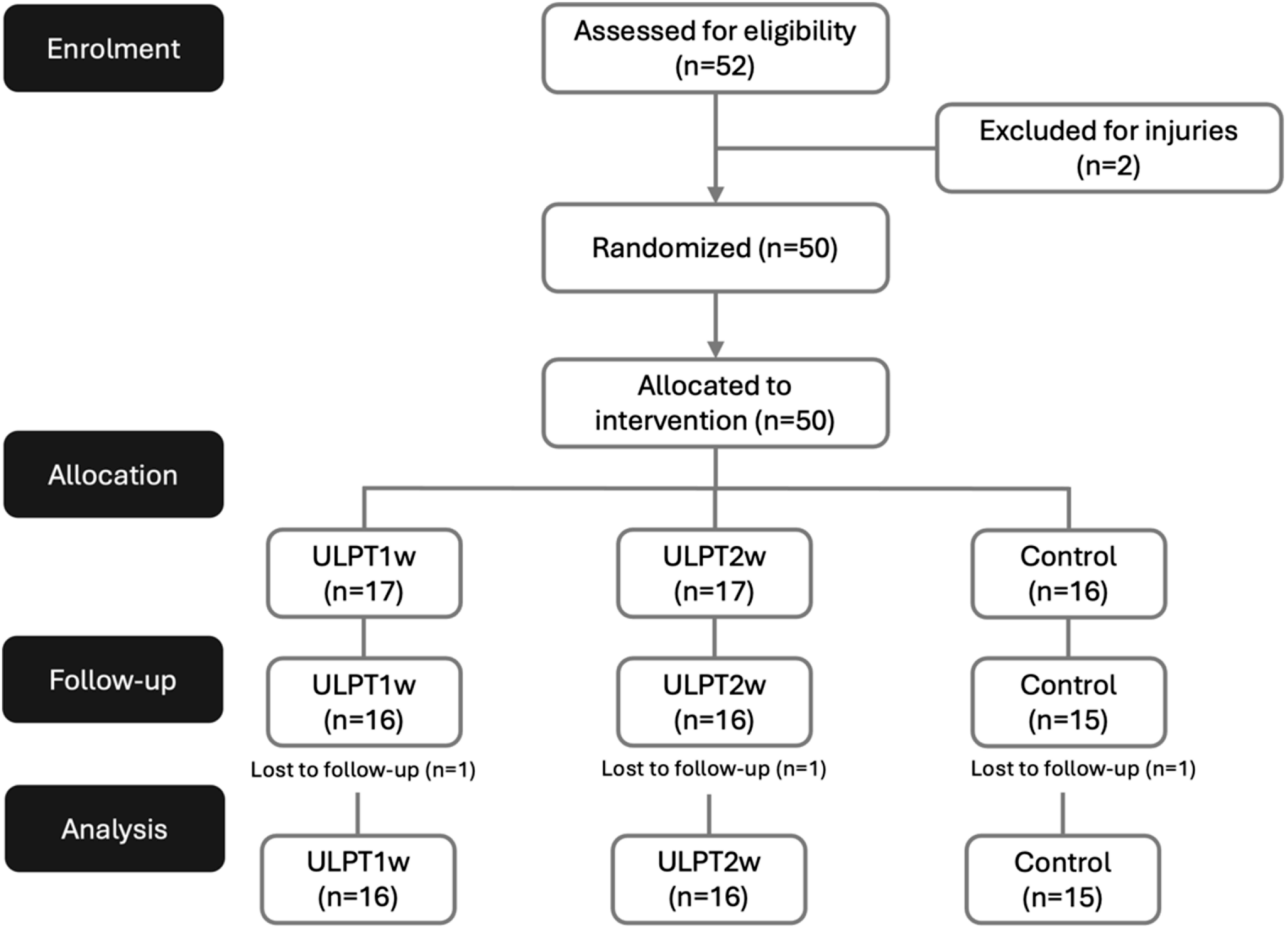
Flowchart of participant progression through the study phases. ULPTw1: group participating in one session per week of upper limb plyometric training; ULPTw2: group participating in two sessions per week of upper limb plyometric training.

Tennis players were qualified for inclusion if they met the following: (i) commitment to attend both evaluation sessions (pre and post-intervention), (ii) at least 4 years of tennis experience, (iii) participation in 85% or more of the intervention sessions, (iv) no injury or illness during the study or in the preceding month, (v) no participation in additional strength or conditioning programs, and (vi) no being previously exposed to a structured ULPT. Conversely, exclusion criteria were defined as (i) missing any evaluation session or test and (ii) using drugs or illegal substances that could influence training adaptations. In total, 52 tennis players were initially enrolled in the study. However, throughout the course of the research, five players were removed from the analysis: two due to injuries from activities not related to the intervention, and three for not attending the first evaluation session (Figure 1).

Ethical approval for the study was granted by the Ethics Committee of Chengdu Institute of Physical Education, with the protocol registered as code number 124 (September 5, 2023). Participants were given comprehensive information about the study’s goals and procedures prior to joining. Informed consent was voluntarily provided by the tennis players parents through signed consent forms as well as the participants. The study followed the ethical guidelines of the Declaration of Helsinki, ensuring voluntary participation throughout.

### 2.2 Interventions

Each group engaged in consistent on-court tennis training, exclusively designed by the coaches, independent of any researcher influence. These sessions typically included warm-up activities that focused on dynamic stretching and footwork drills, followed by strength and conditioning exercises aimed at improving agility and reactivity, combined with technical movements. After that, players would work on individual skill development through drills for forehands, backhands, volleys, and overheads. The session would then transition to set matches or situational drills before concluding with game strategy discussions and formal match play.

In addition to the regular training sessions, the experimental groups—ULPT1w and ULPT2w—also included ULPT. This supplementary training occurred during the first session of the week for both groups. Additionally, the ULPT2w group received ULPT during the third training session of the week (72-h after the first ULPT), which was integrated before their regular training session. All ULPT sessions were supervised by two researchers, both experts in strength and conditioning training, each with over 5 years of experience working with athletes and in sports training. The ULPT was preceded by a standardized warm-up that included 5 min of jogging around the court, followed by dynamic stretching exercises. These exercises comprised arm circles, shoulder dislocations with a resistance band, external and internal rotations using resistance bands, elbow flexion and extension, wrist flexor and extensor stretches, and torso twists, totaling 7 min. After finishing the warm-up, the players were directed to begin the ULPT plan (Table 2).

**Table 2:** Description of the training protocols for the upper limb plyometric training (ULPT).

	ULPT 1^st^ session of the week	ULPT 2^nd^ session of the week
Week 1	2 × 10 Chest throw with 2-kg MB (rest between sets: 2 min) Rest between exercise: 3 min 2 × 10 overhead throw with 2-kg MB (rest between sets: 2 min) Rest between exercise: 3 min 2 × 10 Forehand 2-kg BM Closed Stance (rest between sets: 2 min)	2 × 10 Chest throw with 2-kg MB (rest between sets: 2 min) Rest between exercise: 3 min 2 × 10 overhead throw with 2-kg MB (rest between sets: 2 min) Rest between exercise: 3 min 2 × 10 Forehand 2-kg BM Closed Stance (rest between sets: 2 min)
Week 2	2 × 10 open-stance throw with 2-kg MB (rest between sets: 2 min) Rest between exercise: 3 min 2 × 10 overhead throw with 2-kg MB (rest between sets: 2 min) Rest between exercise: 3 min 2 × 10 2-hand overhead throw with rotation (rest between sets: 2 min)	2 × 10 open-stance throw with 2-kg MB (rest between sets: 2 min) Rest between exercise: 3 min 2 × 10 overhead throw with 2-kg MB (rest between sets: 2 min) Rest between exercise: 3 min 2 × 10 2-hand overhead throw with rotation (rest between sets: 2 min)
Week 3	2 × 10 Chest throw with 2-kg MB (rest between sets: 2 min) Rest between exercise: 3 min 2 × 10 overhead throw with 2-kg MB (rest between sets: 2 min) Rest between exercise: 3 min 2 × 10 Stepping overhead 2-kg MB throw (rest between sets: 2 min)	2 × 10 Chest throw with 2-kg MB (rest between sets: 2 min) Rest between exercise: 3 min 2 × 10 overhead throw with 2-kg MB (rest between sets: 2 min) Rest between exercise: 3 min 2 × 10 Stepping overhead 2-kg MB throw (rest between sets: 2 min)
Week 4	3 × 10 Chest throw with 2-kg MB (rest between sets: 2 min) Rest between exercise: 3 min 3 × 10 overhead throw with 2-kg MB (rest between sets: 2 min) Rest between exercise: 3 min 3 × 10 Forehand 2-kg BM Closed Stance (rest between sets: 2 min)	3 × 10 Chest throw with 2-kg MB (rest between sets: 2 min) Rest between exercise: 3 min 3 × 10 overhead throw with 2-kg MB (rest between sets: 2 min) Rest between exercise: 3 min 3 × 10 Forehand 2-kg BM Closed Stance (rest between sets: 2 min)
Week 5	3 × 10 open-stance throw with 2-kg MB (rest between sets: 2 min) Rest between exercise: 3 min 3 × 10 overhead throw with 2-kg MB (rest between sets: 2 min) Rest between exercise: 3 min 3 × 10 2-hand overhead throw with rotation (rest between sets: 2 min)	3 × 10 open-stance throw with 2-kg MB (rest between sets: 2 min) Rest between exercise: 3 min 3 × 10 overhead throw with 2-kg MB (rest between sets: 2 min) Rest between exercise: 3 min 3 × 10 2-hand overhead throw with rotation (rest between sets: 2 min)
Week 6	3 × 10 Chest throw with 2-kg MB (rest between sets: 2 min) Rest between exercise: 3 min 3 × 10 overhead throw with 2-kg MB (rest between sets: 2 min) Rest between exercise: 3 min 3 × 10 Stepping overhead 2-kg MB throw (rest between sets: 2 min)	3 × 10 Chest throw with 2-kg MB (rest between sets: 2 min) Rest between exercise: 3 min 3 × 10 overhead throw with 2-kg MB (rest between sets: 2 min) Rest between exercise: 3 min 3 × 10 Stepping overhead 2-kg MB throw (rest between sets: 2 min)
Week 7	3 × 8 Chest throw with 4-kg MB (rest between sets: 2 min) Rest between exercise: 3 min 3 × 8 overhead throw with 4-kg MB (rest between sets: 2 min) Rest between exercise: 3 min 3 × 8 Forehand 4-kg BM Closed Stance (rest between sets: 2 min)	3 × 8 Chest throw with 4-kg MB (rest between sets: 2 min) Rest between exercise: 3 min 3 × 8 overhead throw with 4-kg MB (rest between sets: 2 min) Rest between exercise: 3 min 3 × 8 Forehand 4-kg BM Closed Stance (rest between sets: 2 min)
Week 8	3 × 8 Chest throw with 4-kg MB (rest between sets: 2 min) Rest between exercise: 3 min 3 × 8 overhead throw with 4-kg MB (rest between sets: 2 min) Rest between exercise: 3 min 3 × 8 Stepping overhead 4-kg MB throw (rest between sets: 2 min)	3 × 8 Chest throw with 4-kg MB (rest between sets: 2 min) Rest between exercise: 3 min 3 × 8 overhead throw with 4-kg MB (rest between sets: 2 min) Rest between exercise: 3 min 3 × 8 Stepping overhead 4-kg MB throw (rest between sets: 2 min)

MB, medicine ball.

From the first to the third week, the ULPT1w (one training session a week) group performed 60 medicine ball throws per week, while the ULPT2w (two training sessions a week) group completed 120 throws. From the fourth to the sixth week, the ULPT1w group increased their throws to 90 per week, while the ULPT2w group performed 180 throws. During the seventh and eighth weeks, when the intensity was increased by using heavier medicine balls, the ULPT1w group performed 72 throws per week, while the ULPT2w group completed 144 throws. The intervention sessions lasted between 14 and 18 min, including the warm-up.

To ensure that tennis players utilized proper techniques and exerted adequate effort, the two strength and conditioning coaches provided feedback and ensured that every exercise was performed with full intensity to enhance the training effect. Clear instructions were given to players to maximize their effort on each repetition (i.e., maximal intent), with verbal motivation provided during workouts to foster dedication and involvement.

### 2.3 Outcomes

#### 2.3.1 Study design

To prevent specific tennis training from influencing the outcomes, each tennis player was randomly assigned to one of three groups: two experimental intervention groups (ULPTw1 and ULPTw2) and a control group (CON) that continued to their usual tennis training. Twice, evaluations were conducted—before and after the intervention—on the same days of the week to maintain uniform conditions. These assessments occurred indoors in the afternoon. Participants had a 72-h rest period following their last training prior to the evaluations.

Participants for the study were sourced through convenience sampling from a two tennis clubs. To ensure proper randomization and minimize bias from each club’s training approach, the randomization within each club was conducted in a balanced manner, with players being allocated into three groups. Random assignment followed a 1:1 allocation ratio and was implemented by a simple randomization method, involving opaque envelopes given to tennis players prior to their initial assessments, ensuring unbiased allocation. The randomization process was supervised by a researcher uninvolved in later assessments, preserving the blinding procedure.

Independent researchers, with no knowledge of group assignments or intervention details, carried out evaluations 1 week before the intervention, and after 8 weeks of training. Tennis players and coaches, however, were aware of the training protocols being delivered.

#### 2.3.2 Assessments context

The structured sequence of the evaluations began with collecting demographic information and anthropometric measurements. This was succeeded by a warm-up consisting of 5 min of jogging around the court, followed by dynamic stretching exercises. These exercises comprised arm circles, shoulder dislocations with a resistance band, external and internal rotations using resistance bands, elbow flexion and extension, wrist flexor and extensor stretches, and torso twists, totaling 7 min. After the warm-up, participants completed the tests in a predetermined sequence. They began by assessing isometric shoulder rotation strength, measuring both internal (ISRT) and external (ESRT) rotation using a dynamometer. Next, they performed a medicine ball chest throw. Following that, participants engaged in the seated shot-put test (SST) for both their dominant and nondominant arms. Finally, they evaluated serve velocity using a radar gun. To ensure variety, half of the players were randomly chosen to start the unilateral tests (i.e., ISRT, ESRT, and SST right and left) with their left leg, while the other half began with their right leg. This sequence was kept consistent throughout both evaluation sessions. Between each assessment test, a 3-min rest was provided, with a 2-min rest between repetitions within each test. Each player alternated starting with one upper limb, resting, then performing with the other upper limb, and repeating this for the second trial. Each unilateral test included two trials per upper limb during each evaluation, with averages per leg calculated for further analysis. Throughout both evaluation periods, all participants followed the same order and sequence for the assessments.

#### 2.3.3 Isometric shoulder rotation strength

Using a hand-held dynamometer with a 0–500 N range and 0.2 N sensitivity (Nicholas Manual Muscle Test, Co, Lafayette, IN, USA), ESRT and ISRT strength were evaluated. The procedures follow a previous study in tennis players (Moreno-Pérez et al., 2019). Each testing session began with the hand-held dynamometer calibrated according to the manufacturer’s specifications. Participants were placed in a supine position on a bench, ensuring their arms were abducted to 90 degrees and rotated to 0 degrees within the scapular plane. With their elbows flexed at 90 degrees, participants pressed their humerus down against the bench while the testing angle was visually checked.

For measuring ESRT, the hand-held dynamometer calibrated was positioned just proximal to the ulnar styloid process, allowing participants to externally rotate their shoulders against it. In contrast, for assessing ISRT, the hand-held dynamometer was placed just proximal to the radial styloid process, where participants internally rotated their shoulders. To eliminate variability in the process, the dynamometer was secured against a stable, flat surface to prevent any interference from the evaluator.

The assessments for both ESRT and ISRT involved two maximal voluntary contractions lasting 5 s each, with a 30-s rest between sets. Peak strength values were recorded for each repetition, and averages were calculated—both for each limb and across both trials. The results were then normalized according to the participants’ body mass (N/kg).

#### 2.3.4 Medicine ball chest throw

The medicine ball chest throw (MBCT) was conducted following a previous study protocol (Hackett et al., 2018) that showed strong predictive ability for muscular strength and power in adolescents. Participants were seated on an upright bench, approximately at a 90-degree angle. The seat height was adjusted so that their knees were bent at roughly 90 degrees, keeping their feet flat on the floor. They were instructed to push a 3 kg medicine ball away from their chest as forcefully as possible, ensuring that their head, shoulders, and lower back stayed in contact with the bench. A 10-meter tape measure was laid out alongside the bench, and a researcher visually marked where the medicine ball first touched the ground during each throw. The distance achieved was recorded to the nearest 5 cm. Participants were allowed one practice attempts to get accustomed to the movement before starting the actual trials. They then completed two attempts, with 60 s of rest in between, and the longest throw (measured in cm) was registered. Verbal encouragement was provided to motivate them during each trial.

#### 2.3.5 Seated shot-put test

Using a standard 18-inch chair without armrests, the one-arm seated shot put (SST) was conducted, with the front legs aligned on a line marked by the tester (Negrete et al., 2010). An additional 18-inch chair positioned directly in front supported the participant’s feet and lower legs, aligning the hips, knees, and ankles in a straight line parallel to the ground. Across the upper body, a strap was secured diagonally while the non-throwing arm was crossed over the chest (Negrete et al., 2010). A 3-kg medicine ball was utilized, with participants instructed to avoid throwing it in an overhead baseball-style manner. Participants were allowed one practice attempts to get accustomed to the movement before starting the actual trials. Each arm was allowed two attempts, with a 30-s rest period in between. The distance (cm) was measured from the tapeline at the front of the chair to the point where the ball first made contact with the ground. The longest throw (measured in cm) was registered for analysis. Throughout the test, verbal encouragement was provided to support high-intensity effort.

#### 2.3.6 Tennis serve velocity

Measured by a valid and reliable radar gun (model Pocket Radar Ball Coach PR1000BC, Republic of South Korea; Hernández-Belmonte and Sánchez-Pay, 2021), serve speed was set to ‘continuous mode’ to detect maximum ball speed in the range of 40 to 210 km/h. Calibration was completed according to the manufacturer’s specifications prior to each test. Conducted as previous studies (López-Samanes et al., 2017; Moreno-Pérez et al., 2019), the serve test procedure involved positioning the radar in the center of the baseline on the tennis court, 4 meters behind the server, aligned with the approximate height of ball contact (around 2.2 meters) and aimed down the center of the court. Following three practice serves, and three submaximal serves (not registered), the serve velocity test at maximal intent was performed. Required to serve into a designated 1 × 1-meter area in the far diagonal corner of the service area, participants attempted to deliver five maximal speed serves in as few attempts as possible (Moreno-Pérez et al., 2019). Calculated for further analysis was the peak velocity (km/h) of these five serves (Moreno-Pérez et al., 2019).

#### 2.3.7 Reliability of the tests

The coefficient of variation (CV), expressed as a percentage, was calculated within participants for each test, taking into account the repetitions performed within each session. The results indicated a CV of 4.1% for the ISRT and 3.6% for the ESRT, based on the participant averages. The analysis showed a CV of 3.8% for the MBCT, and 4.4% across both arms for the SST. Additionally, the participants’ overall average yielded a CV of 5.2% for tennis serve. The intra-class correlation (ICC) values were used to measure how consistent the test results were. The ISRT had a ICC of 0.85. The ESRT had an even higher ICC of 0.88. The MBCT showed an ICC of 0.86. The SST had a slightly lower ICC of 0.8. Finally, the Tennis Serve test had an ICC of 0.75.

### 2.4 Statistical methods

To assess the normality of the sample, we employed the Kolmogorov–Smirnov test, which yielded *p*-values greater than 0.05. Additionally, Levene’s test was performed to evaluate the homogeneity of variances, also resulting in p-values exceeding 0.05. Comparisons of demographic and anthropometric data between groups at baseline were conducted using one-way ANOVA, followed by the Bonferroni post-hoc test. The CV was used to calculate the variability within each participant across repetitions within the tests. The overall CV was computed as the pooled mean of the individual participant CVs, where the CV for each participant was determined by dividing the standard deviation of their repetitions by the mean value, and then multiplying by 100 to express the result as a percentage. Additionally, the ICC for absolute agreement was calculated. For analyzing the interaction between time and group, a mixed ANOVA was conducted. Effect sizes were calculated using partial eta squared ( $\eta_{p}^{2}$ ) and Cohen’s d for comparisons of pre- and post-intervention measurements. The classification of effect sizes followed the thresholds proposed by Hopkins et al. (2009): small (≥ 0.10), moderate (≥ 0.30), large (≥ 1.2), and very large (≥ 2.0). Post-hoc analyses utilized the Bonferroni test. The JASP software (version 0.18.3, University of Amsterdam, The Netherlands) was used to perform the statistical analysis, with a significance level set at *p* < 0.05.

## 3 Results

Table 3 shows the baseline and post-Intervention performance values for three groups. Significant interactions (time*group) were found in ISRT (*F* = 39.761; *p* < 0.001; $\eta_{p}^{2}$ = 0.644), ESRT (*F* = 32.327; *p* < 0.001; $\eta_{p}^{2}$ = 0.629), MBCT (*F* = 85.933; *p* < 0.001; $\eta_{p}^{2}$ = 0.796), SST (*F* = 95.675; *p* < 0.001; $\eta_{p}^{2}$ = 0.813), and tennis serve velocity (*F* = 43.695; *p* < 0.001; $\eta_{p}^{2}$ = 0.665). No significant differences were found between groups in the baseline for the variables of ISRT (*F* = 0.836; *p* = 0.440; $\eta_{p}^{2}$ = 0.037), ESRT (*F* = 1.652; *p* = 0.203; $\eta_{p}^{2}$ = 0.070), MBCT (*F* = 0.134; *p* = 0.875; $\eta_{p}^{2}$ = 0.006), SST (*F* = 0.010; *p* = 0.990; $\eta_{p}^{2}$ = 0.000), and tennis serve velocity (*F* = 0.999; *p* = 0.376; $\eta_{p}^{2}$ = 0.043).

**Table 3:** Mean and standard deviation (SD) of baseline and post-intervention performance values for three groups.

	ULPT1w (*n* = 16)	ULPT2w (*n* = 16)	Control (*n* = 15)
ISRT (N/kg)			
Baseline	1.80 ± 0.10	1.82 ± 0.13	1.77 ± 0.09
Post-intervention	1.86 ± 0.10^¶^	1.89 ± 0.14^¶^	1.76 ± 0.09
*p*-value and ES (post-pre)	*p* < 0.001; ES = 0.600	*p* < 0.001; ES = 0.519	*p* = 0.211; ES = -0.111
ESRT (N/kg)			
Baseline	1.56 ± 0.03	1.55 ± 0.03	1.57 ± 0.03
Post-intervention	1.61 ± 0.04^¶^	1.61 ± 0.03^¶^	1.57 ± 0.03
*p*-value and ES (post-pre)	*p* < 0.001; ES = 1.429	*p* < 0.001; ES = 2.000	*p* = 0.705; ES = 0.000
MBCT (cm)			
Baseline	621.4 ± 60.5	619.5 ± 61.0	611.5 ± 45.4
Post-intervention	668.1 ± 59.4^¶^	667.1 ± 68.9^¶^	610.2 ± 44.5
*p*-value and ES (post-pre)	*p* < 0.001; ES = 0.779	*p* < 0.001; ES = 0.733	*p* = 0.705; ES = -0.029
SST (cm)			
Baseline	529.3 ± 56.5	531.4 ± 53.1	529.1 ± 42.0
Post-intervention	575.1 ± 57.9^¶^	576.7 ± 50.6^¶^	528.9 ± 43.5
*p*-value and ES (post-pre)	*p* < 0.001; ES = 0.801	*p* < 0.001; ES = 0.874	*p* = 0.923; ES = -0.005
Tennis serve velocity (km/h)			
Baseline	150.8 ± 8.1	152.7 ± 7.2	148.7 ± 8.0
Post-intervention	155.4 ± 8.2^¶^	157.9 ± 7.2^¶^	147.9 ± 8.7
*p*-value and ES (post-pre)	*p* < 0.001; ES = 0.564	*p* < 0.001; ES = 0.722	*p* = 0.122; ES = -0.096

ISRT: internal isometric shoulder rotation strength; ESRT: external isometric shoulder rotation strength; ES: effect size using cohen’s d; SST: one-arm seated shot put test; ULPTw1: group participating in one session per week of upper limb plyometric training; ULPTw2: group participating in two sessions per week of upper limb plyometric training.

¶ Significantly different (*p* < 0.05) from control group.

In the post-intervention, significant differences were observed between groups for the variables of ISRT (*F* = 5.113; *p* = 0.010; $\eta_{p}^{2}$ = 0.189), ESRT (*F* = 6.435; *p* = 0.004; $\eta_{p}^{2}$ = 0.226), MBCT (*F* = 4.871; *p* = 0.012; $\eta_{p}^{2}$ = 0.181), SST (*F* = 4.315; *p* = 0.019; $\eta_{p}^{2}$ = 0.164), and tennis serve velocity (*F* = 6.427; *p* = 0.004; $\eta_{p}^{2}$ = 0.226). Specifically, the control group exhibited significantly lower ISRT values compared to ULPT1w (*p* = 0.014; ES = 1.053, moderate) and ULPT2w (*p* = 0.014; ES = 1.130, moderate). Similarly, the control group exhibited significantly lower ESRT values compared to ULPT1w (*p* = 0.012; ES = 1.143, moderate) and ULPT2w (*p* = 0.008; ES = 1.333, large). In the case of MBCT, the control group had significantly lower values compared to ULPT1w (*p* = 0.026; ES = 1.115, moderate) and ULPT2w (*p* = 0.030; ES = 1.004, moderate), while similarly in SST the control group also presented significantly smaller values compared to ULPT1w (*p* = 0.047; ES = 0.911) and ULPT2w (*p* = 0.038; ES = 0.943). Finally, the control group exhibited significantly lower tennis serve velocity values compared to ULPT1w (*p* = 0.037; ES = 0.888, moderate) and ULPT2w (*p* = 0.004; ES = 1.258, large). No significant differences were observed between ULPT1w and ULPT2w in ISRT (*p* > 0.999; ES = 0.250, small), ESRT (*p* > 0.999; ES = 0.000, trivial), MBCT (*p* > 0.999; ES = 0.016, trivial), SST (*p* > 0.999; ES = 0.029, trivial), and the tennis serve velocity (*p* > 0.999; ES = 0.325, small).

Within-group analysis revealed that ULPT1w showed significant improvements from baseline to post-intervention in the ISRT (*p* < 0.001; ES =0.600, moderate), ESRT (*p* < 0.001; ES = 1.429, large), MBCT (*p* < 0.001; ES =0.779, moderate), SST (*p* < 0.001; ES =0.801, moderate), and tennis serve velocity (*p* < 0.001; ES =0.564, small). Similarly, ULPT2w exhibited significant improvements from baseline to post-intervention in the ISRT (*p* < 0.001; ES =0.519, small), ESRT (*p* < 0.001; ES =2.000, large), MBCT (*p* < 0.001; ES =0.733, moderate), SST (*p* < 0.001; ES =0.874, moderate), and tennis serve velocity (*p* < 0.001; ES =0.722, moderate). In contrast, the control group did not show significant improvements from baseline to post-intervention, as evidenced by the ISRT (*p* = 0.211; ES = −0.111, trivial), ESRT (*p* = 0.705; ES =0.000, trivial), MBCT (*p* = 0.705; ES = −0.029, trivial), SST (*p* = 0.923; ES = −0.005, trivial), and tennis serve velocity (*p* = 0.122; ES = −0.096, trivial).

## 4 Discussion

The current research revealed that both ULPT1w and ULPT2w were effective in enhancing upper limb muscle strength, power, and serve velocity. While both interventions improved muscle strength, power, and tennis serve performance, there were no significant differences between them. Therefore, for young male tennis players who are newly introduced to ULPT, one weekly session may be sufficient to achieve effective adaptations.

The 8-week ULPT training intervention significantly enhanced isometric shoulder rotation strength. It was observed that, regardless of the weekly training frequencies tested, the benefits were equally effective compared to the control group. However, this study does not align with a previous report involving baseball athletes (Carter et al., 2007) who participated in ULPT, which found no statistically significant differences in any of the isokinetic strength measurements between the plyometric and control groups from pre- to post-training. On the other hand, a previous study comparing strength training and ULPT found that the latest one was significantly more effective in enhancing internal rotation (Swanik et al., 2016). Although no studies have specifically examined ULPT in tennis players or analyzed its impact on these tests, a previous study (Behringer et al., 2013) comparing traditional resistance training with plyometric training for the lower and upper limbs found that both programs significantly improved maximal strength, as measured by dynamic exercises like the chest press and pull-down.

ULPT likely fosters neuromuscular adaptations that enhance motor unit recruitment and synchronization, leading to improved force production (McKinlay et al., 2018). The explosive nature of plyometric exercises may have stimulated muscle power development through the stretch-shortening cycle, which can translate into increased isometric strength (Walshe et al., 1998). Additionally, adaptations in tendon stiffness and strength may contribute by enhancing force transmission (Ramírez-delaCruz et al., 2022). Even with varying training frequencies, the intensity of the exercises provided a sufficient stimulus for muscle adaptations. This effect can be further justified by the youth of the players, as younger athletes tend to exhibit higher trainability and a greater capacity for adaptation, making them particularly responsive to this type of training (Michailidis et al., 2013).

Both the MBCT and the Seated Shot-Put Test showed significant and positive improvements after the ULPT compared to the control group, with no significant differences observed among the training frequencies in which the players participated. The results align with a study by Fernandez-Fernandez et al. (2016) which reported significant benefits of introducing ULPT for enhancing the MBCT. Additionally, they are consistent with findings from another study that demonstrated positive adaptations in MBCT following plyometric training (Fernandez-Fernandez et al., 2018). A possible explanation for observing these positive adaptations is that ULPT may significantly enhanced the neural and physiological mechanisms by optimizing the stretch-shortening cycles and potentiation, which may have activated the stretch reflex and increased neural drive to the muscles involved in throwing (Turner and Jeffreys, 2010). This process possibly facilitated greater recruitment of fast-twitch muscle fibers, known for their ability to produce high force and power output quickly (Macaluso et al., 2012). Additionally, plyometrics possibly improved neuromuscular efficiency by enhancing motor unit synchronization and firing rates, leading to more coordinated and forceful muscle contractions (Wallace and Janz, 2009). These adaptations may result in heightened muscle stiffness and elasticity, which contributed to a more effective transfer of force during explosive movements (Fouré et al., 2011).

The serve speed improved in both ULPT groups, regardless of training frequency, and was significantly more effective compared to the control group. This finding aligns with a previous study (Fernandez-Fernandez et al., 2016) in adolescent tennis players, which found that after combining ULPT with lower limb plyometric training, serve velocity increased by 6.2%, a significant improvement over the control group. Our results also align with another study (Terraza-Rebollo et al., 2017) that showed an 8-week program using resistance, medicine balls, and elastic bands significantly enhanced both serve speed and medicine ball throwing ability in adolescent tennis players. Additionally, our findings are in agreement with a study in adolescent tennis players (Behringer et al., 2013) that compared upper and lower limb plyometric training with strength training and a control group, revealing that plyometrics alone led to a unique increase in serve velocity, by approximately 3.8%. The possibly improved mechanism of the stretch-shortening cycle may have enabled muscles to store elastic energy during a quick pre-stretch phase, releasing it explosively during the subsequent contraction phase (Turner and Jeffreys, 2010). This rapid cycle possibly enhanced the speed and power of upper limb movements by increasing both neural activation and muscle-tendon elasticity (Swanik et al., 2016), which are essential for producing forceful and fast serves. Furthermore, the tennis serve relies heavily on powerful actions (Colomar et al., 2023), which showed significant improvement through MBCT and the Seated Shot-Put Test. These enhancements support the observed increase in serve velocity, aligning with previous studies that showed the interdependence of these muscle power measures and serve performance (Palmer et al., 2018).

The lack of observed differences between ULPT1w and ULPT2w may be attributed to the heightened responsiveness of adolescent muscle fibers to neuromuscular adaptations, driven by growth-related increases in muscle size and hormonal changes (Gillen et al., 2019). Plyometric training can optimally support these physiological developments, enhancing neuromuscular efficiency and coordination (De Almeida et al., 2021). Research suggests that neural and muscular adaptations are particularly pronounced in younger athletes (Legerlotz et al., 2016), making plyometric training especially beneficial for adolescent tennis players. This age group’s greater trainability, combined with their first exposure to this type of training, may have enabled significant improvements even with just one session per week.

Despite the positive findings, this study has some limitations that should be addressed in future research. The sample consisted solely of young male tennis players, limiting the generalizability of the findings to other ages, trainability levels, and genders. Future studies should increase diversity by including older athletes and female participants to better assess ULPT’s broader applicability. Additionally, the 8-week duration does not account for potential long-term effects, making it essential to examine ULPT’s sustainability and optimal training frequencies and duration. This could help identify any performance plateaus or phases of increased sensitivity to training volume adjustments. Another limitation is the absence of a strain gauge capable of recording the force-time curve, due to resource constraints. However, this approach would provide valuable insights into a critical aspect of the analysis, and future studies should incorporate it. Furthermore, incorporating biomechanical analyses, such as motion capture or electromyography, could provide a deeper understanding of the neuromuscular adaptations associated with ULPT, which were not explored in this study. Finally, our study may also have a limitation in that we replicated the same exercises in the second session of the week as those implemented in the first. Including different exercises in the second session could potentially yield different results, and this should be acknowledged and explored in future studies.

Despite the limitations, the findings of this study offer practical implications for coaches working with young male tennis players. Given that both one and two weekly ULPT sessions effectively enhanced upper limb strength, power, and serve velocity, it appears that a single weekly session may be sufficient to produce meaningful adaptations in beginners. This is particularly valuable for time-constrained training schedules, allowing players to allocate more time to other skill-based without compromising upper limb development. Additionally, the study highlights the utility of ULPT in fostering muscular power adaptations crucial for explosive and determinant movements like serving. By incorporating ULPT into training, coaches can help players optimize upper limb performance, which is critical for effective serve velocity and overall competitive performance.

## 5 Conclusion

The study concluded that ULPT is an effective training intervention for improving upper limb muscle strength, power, and serve velocity in young male tennis players. Both ULPT training frequencies (once and twice weekly) showed significant performance enhancements over the control group, with no meaningful differences between the two frequencies, indicating that a single weekly session may suit for positive adaptations in youth players. Despite promising results, the study is limited by its focus on young males, making further research necessary to explore effects across different ages, genders, and long-term outcomes. Coaches can utilize these findings to implement ULPT one a week in training, even on restricted schedules, to promote upper limb power and serve speed, essential for competitive tennis performance.

## Data Availability

The raw data supporting the conclusions of this article will be made available by the authors, without undue reservation.
